# Post-operative atrial fibrillation after cardiac surgery: Challenges throughout the patient journey

**DOI:** 10.3389/fcvm.2023.1156626

**Published:** 2023-03-07

**Authors:** William F. McIntyre

**Affiliations:** Population Health Research Institute and Department of Medicine, McMaster University, Hamilton, ON, Canada

**Keywords:** atrial fibrillation, cardiac surgery, stroke, rate control, rhythm control, POAF

## Abstract

Atrial fibrillation (AF) is the most common complication of cardiac surgery, occurring in up to half of patients. Post-operative AF (POAF) refers to new-onset AF in a patient without a history of AF that occurs within the first 4 weeks after cardiac surgery. POAF is associated with short-term mortality and morbidity, but its long-term significance is unclear. This article reviews existing evidence and research challenges for the management of POAF in patients who have had cardiac surgery. Specific challenges are discussed in four phases of care. Pre-operatively, clinicians need to be able to identify high-risk patients, and initiate prophylaxis to prevent POAF. In hospital, when POAF is detected, clinicians need to manage symptoms, stabilize hemodynamics and prevent increases in length of stay. In the month after discharge, the focus is on minimizing symptoms and preventing readmission. Some patients require short term oral anticoagulation for stroke prevention. Over the long term (2–3  months after surgery and beyond), clinicians need to identify which patients with POAF have paroxysmal or persistent AF and can benefit from evidence-based therapies for AF, including long-term oral anticoagulation.

## Introduction

Each year, more than a million adults undergo cardiac surgery in North America and Europe; this number is expected to increase along with an aging population and a growing burden of co-morbidities (1–3). Postoperative atrial fibrillation (AF) is the most common complication after cardiac surgery, occurring in up to 50% of patients (4). POAF is associated with adverse outcomes and poses significant management challenges to clinicians.

Patients who develop POAF have strokes at roughly four times the rate as those who do not and twice as likely to die (4–6). Patients with POAF spend longer in hospital, including up to 2 days more in the intensive care unit and an additional 3 days the hospital overall (5–8). Moreover, they are 30% more likely to be readmitted to hospital in the month after surgery (5–8). In the early years following surgery, patients who had POAF experience a 2-to-4 fold increase in their risk of stroke and a 25% increase in the risk of death (5, 9).

**Figure 1:**
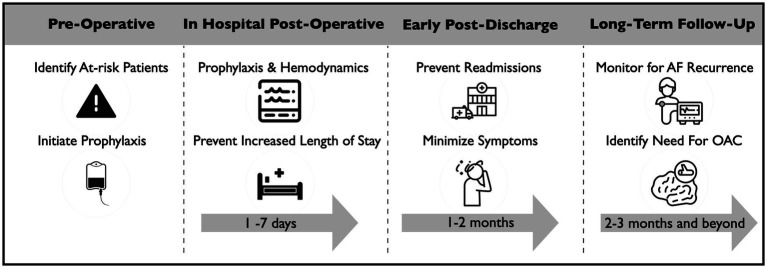
Management challenges for patients with POAF after cardiac surgery.

Despite the prevalence and impact of POAF, there is marked uncertainty and practice variability with respect to its prevention and treatment. This article defines POAF and reviews the main clinical challenges and research questions for patients with POAF across the peri-operative patient journey (Figure 1).

### What is post-operative atrial fibrillation?

POAF refers to new-onset AF (i.e., in a patient without a prior history of the arrythmia) occurring early enough after surgery that there is uncertainty as to whether it is a ‘reversible’ entity that was “caused” by surgery or paroxysmal/persistent AF that was first detected around the time of surgery (10).

POAF after cardiac surgery is understood to have a complex pathophysiology. Acute factors (e.g., inflammation, adrenergy, vasoactive medications, ischemia, metabolic disturbances, volume overload, operative manipulation of the heart) and fixed substrate (e.g., valvular disease, atrial myopathy, hypertension) are thought to interact to trigger arrhythmia (11, 12). Studies using continuous ECG monitoring have described episodic increases in AF in the post-operative period, beyond background rates. These studies have shown that the incidence and prevalence of POAF peak around the second to third post-operative day and then decrease, leveling off in the third and fourth weeks after surgery (13–15). In a study by Bidar et al., the prevalence of AF reached 5% on the first post-operative day and stayed at this level until the 16th post-operative day, whereafter it remained around 2% for the 14 remaining days of follow-up (14). In the SEARCH-AF trial, the overall incidence of AF did not increase beyond the fourth week of continuous ECG monitoring (15). Based on these patterns, AF that occurs within 28 days post-operatively should be classified as post-operative AF, and of uncertain long-term significance. In contrast, AF occurring after 28 days post-operatively should considered to be paroxysmal or persistent AF, rather than a potentially “reversible” form of AF. For patients with POAF, clinicians have many challenges. They are tasked with preventing the arrhythmia in at-risk patients, mitigating its negative effects in the months after surgery, and identifying patients who have AF recurrence beyond 28 days post-operatively to match them with evidence-based therapies for the general AF population.

### Prevention and prediction of POAF

Researchers have devoted significant resources to developing models to predict POAF and to identifying safe and effective therapies to prevent it. Accurate prediction is desirable to identify the highest risk patients for both intensive monitoring and targeted prophylaxis.

Dozens of papers have been published assessing different risk scores for predicting POAF. Most of these papers use scores that were developed for other purposes [e.g., the EuroScore for predicting mortality after cardiac surgery, (16) or the CHADS-VASc score for predicting stroke risk in AF patients (17)] and perform only modestly. A dedicated POAF score (incorporating age, chronic obstructive pulmonary disease, emergency operation, preoperative intra-aortic balloon pump, left ventricular ejection fraction <30%, estimated glomerular filtration rate < 15 mL/min/m^2^ or dialysis and valve surgery) was published in 2014 but has not been widely implemented in clinical practice nor in subsequent research (18). There is a clear need for improved performance and implementation of risk scores. Scores that leverage a wide variety of clinical and surgical characteristics and modern tools such bio-informatics (including polygenic risk scores, proteomics, biomarkers) and artificial intelligence may offer the best prediction and discrimination.

A number of therapies have been tested for the prophylaxis of POAF. Among these, beta-blockers have some of the most robust evidence. A 2019 Cochrane systematic review including 63 randomized trials concluded that beta-blockers reduced the risk of POAF (relative risk (RR) 0.50, 95% CI, 0.42–0.59; *I*^2^ = 59%) (19). However, the evidence was rated as low certainty due to several studies being at high risk of bias and a moderate level of statistical heterogeneity that was not explained through subgroup analysis. Moreover, there was uncertainty about safety with wide confidence intervals around effect estimates for hypotension and stroke, and concerns that the evidence was rated as moderate due to several studies being at high risk of bias. Clinical practice guidelines from the Canadian Cardiovascular Society (CCS), European Society of Cardiology (ESC), and European Association for Cardiothoracic Surgery (EACTS) all recommended perioperative beta-blocker therapy to prevent POAF after cardiac surgery (12, 20, 21). Despite these data and guideline recommendations, beta-blockers are not always used for prophylaxis. A survey conducted by the Society of Cardiovascular Anesthesiologists (SCA) and the European Association of Cardiothoracic Anesthetists (EACA) found divergent practices with respect to the perioperative use of beta-blockers (22). The proportion of providers who said that they nearly always followed the guideline recommendations for beta-blocker prophylaxis was 43%, whereas 36% reported that they followed the guideline sometimes, and 21% reported that they rarely follow the guidelines. This survey found that the perception that the risks of hypotension outweighed the benefits was the most common reason for non-use of beta-blockers. Landiolol is an ultra-short acting beta-1 selective beta blocker that is used in Japan and in some European countries and is currently under investigation elsewhere (23, 24). Landiolol may offer effective prophylaxis against POAF without adverse hemodynamic effects.

Amiodarone has been proven in trials to be effective for the prevention of POAF (25, 26). However, the SCA/EACA survey also established that it is not widely used, due to risk of side effects and need for loading doses beginning days in advance of surgery (22). A comprehensive 2013 Cochrane Review of interventions for POAF prophylaxis identified 118 studies with 138 treatment groups and 17,364 participants (26). In addition to efficacy for beta-blockers and amiodarone, the review found a reduction in POAF associated with each of the other studied interventions, including: sotalol, magnesium, atrial pacing and posterior pericardiotomy. Interventions were associated with shorter lengths of stay and reduced cost. Several other approaches are under investigation, including botulinum toxin and neuro-modulation (27, 28). Successful strategies will require demonstration of safety and efficacy in addition to ease of administration and knowledge translation.

### In-hospital treatment of POAF

The 25–50% of cardiac surgery patients who develop POAF are in need of therapy to prevent short term-consequences. During this phase of care, the main goals are minimizing symptoms, stabilizing hemodynamics and preventing increases in hospital length of stay. For these patients, clinicians have a choice of two broad strategies: rhythm control and rate control. Rhythm control focuses on restoring and maintaining sinus rhythm with anti-arrhythmic drugs or electrical cardioversion. In contrast, rate control uses one or more negative chronotropic drugs to control the ventricular rate. We conducted a systematic review, finding 8 randomized trials that included 990 patients and concluded that evidence suggests no clear advantage to either rhythm or rate control for length of stay, AF recurrence or mortality (29). We judged the quality of evidence to be low due to risk of bias and imprecision. In these studies, AF recurrence was ascertained using single resting 12-lead ECGs. The dominant trial in this review was published 2016 by Gillinov et al. (30). That study enrolled 2,109 patients pre-operatively, and from the 695 (33.0%) who developed POAF, they randomized 523 patients to an *initial* rate or rhythm control strategy. The recommended dose of amiodarone was the equivalent of 3 g of oral amiodarone before hospital discharge, with a maintenance dose of 200 mg per day for 60 days. The authors found no difference in the primary outcome of number of hospital days at 60 days follow-up (median, 5.1 days and 5.0 days *p* = 0.76) nor in the rates of overall serious adverse events (24.8 per 100 patient-months in the rate-control group and 26.4 per 100 patient-months in the rhythm-control group, *p* = 0.61). The authors did demonstrate that continued freedom from AF, as ascertained using 12-lead ECGs at days 0, 30 and 60, was more common in the rhythm control group, as compared to the rate control group (97.9% vs. 93.8%. *p* = 0.02). This trial has important limitations, which may have led to it showing no difference in length of stay. Because this trial enrolled patients before the development of AF, the population included a mix of high and low risk (e.g., those with very short durations of AF) patients; this may have obscured clinical benefit. Additionally, the rate of cross-over between arms was extreme, reaching 25% in both groups. Cross-overs were driven by lack of efficacy in the rate control arm and side effects in the rhythm control arm. Overall, this trial showed a high use of rhythm control among patients with POAF and that amiodarone therapy reduces AF. However, questions regarding the efficacy of a rhythm control to reduce clinical outcomes remain because of limitations of the study. Among major international guidelines published after this trial, only the 2017 EACTS Guidelines and the 2020 CCS Guidelines mention this trial directly (12, 20, 21, 31, 32). The 2020 CCS Guidelines simply recommend that “*AF after cardiac surgery may be appropriately treated with a rate control strategy or a rhythm control strategy*” (Strong Recommendation; Moderate-Quality Evidence) (12). In contrast, the 2017 EACTS Guidelines appraised the trial, noting the above limitations, and recommended “*rhythm control in patients with hemodynamically stable POAF*” (Class I, Level B) and stated that “*rate control can be considered in hemodynamically stable, asymptomatic patients*” (Class IIa, Level B) (20). The current guidelines leave considerable flexibility for clinicians to tailor therapy to patient needs. A larger randomized trial that focuses on higher risk patients and minimizes cross-overs will help clarify for what group of patients with POAF, if any, rhythm control is effective.

### Short-term (first 1–3 months) therapy post discharge

Patients with POAF after cardiac surgery are 30% more likely to be readmitted to hospital in the month after surgery (5–8). Despite the uncertainty in efficacy as outlined in the previous section, rhythm control remains the most-used strategy during this phase of care. A report of 166,747 patients who developed POAF after CABG (incidence 24%) in the Society of Thoracic Surgeons Adult Cardiac Surgery Database from 2011 to 2018 showed that more than three-quarters were discharged on amiodarone (33). Despite the frequency of its use, there is little guidance on *how* to provide rhythm control of AF after cardiac surgery. Clinical practice guidelines recommend amiodarone for at least 4 weeks when rhythm control is chosen. However, these documents do not cite any primary evidence to support these recommendations. The CCS Guidelines suggest 6–12 weeks of therapy with amiodarone (12, 34, 35). The ESC/EACTS guidelines suggest 4 weeks of therapy with amiodarone (20, 21, 36). The American Association of Thoracic Surgeons (AATS) guidelines suggest 4–6 weeks of therapy with amiodarone (37). Among these, only the AATS guideline provides dosing recommendations: 150 mg IV over 10 min; then 1 mg/min infusion for 6 h; then 0.5 mg/min IV continuous infusion for 18 h or change to oral administration at 100–400 mg daily. Amiodarone has unique pharmacokinetics. Due to its cationic and amphiphilic profile, it is trapped in body tissues and then released slowly into the plasma. Thus, therapeutic plasma concentrations of amiodarone can persist for weeks after dosing has stopped (38). Therefore, a shorter course of amiodarone may be sufficient to provide plasma concentrations to counteract the front-loaded pro-arrhythmic process that occur following surgery. A shorter amiodarone regimen has three potential advantages. First, patients and physicians are often hesitant to use ongoing amiodarone because of its potential side effects (39). Patients who receive amiodarone frequently stop their medication prematurely (25). In the Gillinov trial of rate versus rhythm control, 15–20% of amiodarone users abandoned therapy early due to side effects (30). Although amiodarone toxicity from short-term use is uncommon, short courses can disturb thyroid function and rarely result in acute respiratory distress syndrome (40–43). A shorter course of amiodarone may enable compliance while minimizing side effects. Second, amiodarone interacts with many drugs that are commonly used after cardiac surgery, including warfarin, statins and calcium channel blockers. Observational data suggest higher rates of bleeding in patients receiving amiodarone and oral anticoagulation (OAC) compared to OAC alone (33). Finally, for some patients, POAF is the first manifestation of paroxysmal AF, while in others the arrhythmia can be considered a reversible complication of surgery. The simplest way to make this distinction is for the patient to undergo continuous ambulatory ECG monitoring after they have recovered from surgery. Amiodarone confounds this assessment. With a shorter course of amiodarone, this assessment can be done earlier, which could limit unnecessary exposure to OAC and allows patients to be selected for therapies to prevent symptoms and progression of AF. Given the predominance of rhythm control with amiodarone and the limited data that inform *how* it should be provided, generating evidence on optimal use of rhythm control is a priority.

The role of short-term oral anticoagulation for stroke prevention in patients with POAF is unclear. The risk of bleeding is higher close to surgery and much of the AF is thought to be transient (6). The open-label, randomized Anticoagulation for New-Onset Post-Operative Atrial Fibrillation After CABG (PACES) trial (NCT04045665) is assessing the safety and efficacy of OAC in this population.

### Long-term therapy (2–3  months and beyond) post discharge

POAF is thought to be a reversible entity in a large proportion of patients (4, 20, 21). However, an important subset of patients with POAF have paroxysmal or persistent AF and can benefit from long-term OAC for stroke prevention. Proven strategies are needed to help identify this group of patients. In POAF patients, AF frequently disappears as patients recover from surgery. What is not known is to what degree patients who manifest POAF have a propensity for future development of AF. This uncertainty affects the view of whether or not a patient with POAF can be considered to have true, “clinical” AF and therefore the accompanying risks of stroke, mortality and heart failure and likewise the propensity to respond to established treatments for AF. Perception of the risk of AF recurrence after an episode of POAF affects how patients are managed in terms of rhythm monitoring and prescriptions, including thromboprophylaxis. In the early years following surgery, patients who had POAF carry a 2-to-4-fold increase in their risk of stroke and a 25% increase in the risk of death (5, 9). This population’s absolute event rates are higher than in controls without AF, but lower than in patients with the common form of AF. The subset of patients with POAF who are at long-term risk for adverse events are expected to have recurrent AF episodes. The 2020 ESC AF Guidelines stated: “*Long-term OAC therapy to prevent thromboembolic events may be considered in patients at risk for stroke with postoperative AF after cardiac surgery, considering the anticipated net clinical benefit of OAC therapy and informed patient preferences*.” (21). However, this is only a Class IIb recommendation, with level B evidence. The 2020 CCS guidelines recommend that “*patients who have experienced AF after cardiac surgery be followed indefinitely for the possible emergence of recurrent clinical AF.”* However, they do not give any recommendations on how to do this. The 2014 American College of Cardiology/American Heart Association/Heart Rhythm Society guidelines acknowledged that we are lacking in data to direct the long-term management of patients with POAF (44).

Some data exist on the recurrence of AF following POAF, but studies are either limited by retrospective designs relying on opportunistic diagnosis of AF or use of implantable lop recorders (ILRs), a tool that is impractical for routine clinical practice. A prospective study of 80 patients who were enrolled in a randomized trial of left atrial appendage occlusion found that over a mean follow-up of 3.7 ± 1.6 years, AF recurrence, as documented in electronic hospital medical records, occurred in 43.8% of patients (45). Patients with POAF had 12.24 times higher hazard ratio for AF during follow-up (95% CI 4.76–31.45, *p* < 0.001) as compared to the group without POAF. In a 73,543-person retrospective cohort study of cardiac surgical patients using administrative data, recurrence of AF after discharge, as ascertained through health care claims codes was 22.2% at one-year (9). This was significantly higher than the rate of AF in patients without POAF (4.7%). At least three small studies have used ILRs to monitor for AF recurrence beyond 1 month in patients with POAF following cardiac surgery (13, 46, 47). The number of patients with POAF ranges from 23 to 42 and AF recurrence rates range from 33 to 76%. Together, these data establish that AF recurrence after POAF could be common. There is however, very little evidence using ECG monitors that can be widely implemented in clinical care; ILRs are invasive and cost several thousand dollars per patient and are thus impractical for use in routine clinical care. Another important limitation is that the ILR studies of POAF patients did not include a comparator group. In the ASSERT-II study, 256 non-surgical patients without a history of AF received an ILR and were followed for 16.3 ± 3.8 months, AF ≥5 min was detected in 34.4% of participants (95%CI, 27.7–42.3) (48). This is comparable to the rates of AF that were seen in the small ILR studies of POAF patients. Thus, a comparator group is needed to show that the finding of increased AF is unique to the POAF population. Prospective studies are needed to better define the recurrence rate of AF after POAF. These studies should use systematic follow-up with contemporary continuous ECG technology and a control population that is free of POAF. These studies should also be large enough to explore predictors of AF recurrence, including left atrial size, which has been identified as a strong predictor of AF in other populations (48, 49).

### Summary

Atrial fibrillation (AF) is the most common complication of cardiac surgery, occurring in up to half of patients. Post-operative AF (POAF) refers to AF in a patient without a history of AF that occurs within the first 4 weeks days after cardiac surgery. POAF occurs after 25–50% of cardiac surgeries, and associated with short-term mortality and morbidity, but its long-term significance is unclear. POAF brings unique challenges at different points in the patient journey.

Pre-operatively, clinicians need to be able to identify high-risk patients, and initiate prophylaxis to prevent POAF. In hospital, when POAF is detected, clinicians need to manage symptoms, stabilize hemodynamics and prevent increases in length of stay. In the month after discharge, the focus is on minimizing symptoms and preventing readmission. Some patients require short term oral anticoagulation for stroke prevention. Over the long term (2–3 months after surgery and beyond), clinicians need to identify which patients with POAF have paroxysmal or persistent AF and require long-term oral anticoagulation and/or rate and rhythm control. Each phase of care is in need of further research to guide clinical care (Box 1).

Box 1. **Key Clinical Problems and Knowledge Needed to Improve the Care of Patients with Post-operative AF After Cardiac Surgery**

**Table 1:** 

**Pre-Operative**	**Clinical problems** Prophylactic strategies are not consistently implemented Available prediction tools perform modestly **Knowledge needed** Improved prophylactic strategies, coupled with implementation and knowledge translation Improved prediction leveraging artificial intelligence and bio-informatics in addition to clinical characteristics
**Early Post-Operative**	**Clinical problems** POAF is associated with longer intensive care stay, hospital stay and higher health-care costs **Knowledge needed** Defining which patients will benefit from a rhythm or rate control strategy, ideally in a randomized trial that focuses on high-risk patients and minimizes cross-overs
**Early Post-Discharge** **(1–2 months)**	**Clinical problems** Patients with POAF are 30% more likely to be readmitted to hospital. Amiodarone is the dominant treatment strategy but the optimal treatment regimen is unclear POAF may pose a short-term, possibly transient risk of stroke **Knowledge needed** Defining the optimal amiodarone treatment regimen that reduces the risk of symptomatic AF recurrence while minimizing side effects and permitting timely stratification of long-term risk Establishing the safety and efficacy of oral anticoagulation in the early post-operative period
**Late Post-Discharge** **(2–3 months and beyond)**	**Clinical problems** A subset of patients with POAF have paroxysmal or persistent AF and require long-term therapy, including oral anticoagulation, while others do not **Knowledge Needed** Defining the recurrence rate of AF in POAF patients using widely available clinical monitors, ideally in a prospective study with systematic follow-up, an appropriate comparator group and exploration of risk factors for AF recurrence

## Author contributions

The author confirms being the sole contributor of this work and has approved it for publication.

## Conflict of interest

The author declares that the research was conducted in the absence of any commercial or financial relationships that could be construed as a potential conflict of interest.

## Publisher’s note

All claims expressed in this article are solely those of the authors and do not necessarily represent those of their affiliated organizations, or those of the publisher, the editors and the reviewers. Any product that may be evaluated in this article, or claim that may be made by its manufacturer, is not guaranteed or endorsed by the publisher.
